# Investigation of the Evaporation Rate of Water from Colloidal Unimolecular Polymer (CUP) Systems by Isothermal TGA

**DOI:** 10.3390/polym12112752

**Published:** 2020-11-21

**Authors:** Peng Geng, Ashish Zore, Michael R. Van De Mark

**Affiliations:** Department of Chemistry, Missouri S&T Coatings Institute, Missouri University of Science and Technology, Rolla, MO 65409, USA; pgkr4@mst.edu (P.G.); aszbnd@mst.edu (A.Z.)

**Keywords:** colloidal unimolecular polymer (CUP), nanoparticle, evaporation rate, thermogravimetric analyzer (TGA), counterion condensation, diffusion, deformation

## Abstract

Studies of the evaporation of aqueous nanoparticle solutions have been limited due to lack of homogeneity of the solution, difficulties in obtaining reproducible samples and stability of substrates, as well as the effect of other volatile components or contaminants such as surfactants. Colloidal unimolecular polymer (CUP) is a spheroidal nanoparticle with charged hydrophilic groups on the surface, and the particle size ranges from 3 to 9 nm. The large amount of surface water on the CUP surface provides the opportunity to evaluate the evaporation of surface water, which may contribute to the investigation the factors that affect the evaporation rate in solutions of ultra-small particles, like protein, micelle, colloidal, etc. Six CUP systems were evaluated by thermogravimetric analysis (TGA) with respect to time and solids content. The evaporation rate of water was initially enhanced due to the deformation of the air-water interface at low to moderate concentration due to particle charge repulsive forces. At higher concentrations, above 20%, surface charge condensation and increasing viscosity began to dominate. At higher concentration where the CUP reached the gel point the rate of diffusion controlled the evaporation. The final drying point was the loss of three waters of hydration for each carboxylate on the CUP surface.

## 1. Introduction

In past decades, the evaporation of aqueous nanoparticle solutions has been a topic of interest, and it is one of the most important, fundamental kinetic and thermodynamic characteristics, which offers an opportunity to investigate the basic concept in diffusion, surface behavior, polymer properties and solution dynamics [1,2]. In addition, the investigation of the water evaporation of aqueous nanoparticles solutions provide a great study model for DNA packing, protein drying processes and drug delivery, also its potential application in the drying of water borne coatings, water borne pesticides and biocides, herbicide, cosmetics, and many others [3,4,5,6,7]. The evaporation rate of water has many significant economic impacts from efficacy for crop protection to drug production rates and even the drying of water borne coatings.

Boukherroub et al. [8] reported an increase in the evaporation rate of water-based graphene nanofluids. It was proposed that graphene oxide functionalization with polyethylene glycol promoted the dispersion of graphene nanoparticles and increased the evaporation rate at constant temperature. The potential agglomeration and poor dispersion of graphene nanoparticles at high concentration could cause a decrease in the evaporation rate. Kim et al. [9] found that the evaporation rate of nanofluid aqueous droplet was higher than deinoized water with the presence of 80 nm sized CuO powder under the same experimental condition. The increase in the evaporation rate was considered to be caused by the nanofluid having better thermal conductivity. Aslani et al. [10] investigated the evaporation rate of water from clay particles in aqueous solution under isothermal condition, with a particle size ranging from 25 to 30 nm. The experiment involved dispersion of nano-sized powder using an ultrasonic processor, and conducted by putting a heating vessel on a digital scale, which were all placed inside a wind tunnel to provide changing velocity. It was found that these particles were able to reduce the surface tension and therefore increased the evaporation rate, and with the increase of concentration, the evaporation efficiency was enhanced. In general, most studies on evaporation rate were performed with metal oxide/metal nanoparticles, nanostructures of carbon and non-charged nanoparticles. 

Most solution/suspension used to study evaporation rate lacks homogeneity and is difficulty to obtain reproducible samples [11]. Another major issue is the lack of stability of substrates. When the percent solids reaches a high level, most nanoparticles tend to aggregate due to Van der Waals forces [12], and change the microstructure or configuration [13,14]. 

Colloidal unimolecular polymer (CUP) is a spheroidal nanoparticle with charged hydrophilic groups on the surface, and particle size ranging from 3 to 9 nm depending on the molecular weight [15]. Several advantages make CUP an ideal system to conduct evaporation studies. The surface area to volume ratio is ultra-high due to the small particle size, which significantly enhanced the properties of their aqueous solutions, like viscosity, surface tension, etc. [16,17,18]. CUPs can be easily synthesized and obtained through a water reduction process [19]. The surface charge density and molecular weight of CUP can be designed to fit a need and the surface structure is predictable and reproducible. CUP particles, once formed, are thermodynamically stable solutions in water, and can be dried and re-dissolve in water without aggregation. Therefore, CUPs are able to show the detailed process of how surface water is released in the drying process. Furthermore, CUP particles have charged hydrophilic groups on the surface that can associate with a large amount of surface water [20]. With the small particle size, CUPs offer an ultra-high surface water fraction [21], and therefore could significantly magnify the observation of the surface waters contribution in the evaporation process. In addition, CUP systems are truly zero VOC with no additives. Thus, CUP is considered an ideal particle to investigate the effect of charged nanoparticles and their associated surface water on evaporation. 

TGA is the most common technique used for mass change, kinetic analysis [22,23,24,25]. This technique is based on the evaluation of mass loss of the studied sample in a specific gas stream at a given temperature or programmed temperature [26]. TGA allows for a small sample size while giving precise measurement of the mass change. The constant continuous flow of inert gas can maintain the stable experimental condition and minimize the formation of thin moisture layer above the aqueous surface that may reduce the evaporation rate. TGA is considered a very appropriate instrument for this study. 

This manuscript presents a primary study of the behavior of free water and surface water in CUP systems during the evaporation of the water. The evaluation of molecular weight and surface charge density, in ions per nm^2^, effects on the evaporation rate was quantified by TGA. A packing model for CUP particles during the evaporation process of water was proposed. The aim was to develop the knowledge of possible factors that affect the evaporation rate of CUP solutions so as to offer fundamental insight for how to design CUP particles which give the best properties for a given application, avoiding a trial-and-error approach.

## 2. Materials and Methods 

### 2.1. Materials and Synthesis

CUP particles used in this paper were synthesized, characterized, and formed into CUP particles and were reported in our earlier report [27]. Table 1 gives the critical data for these polymers.

### 2.2. Thermogravimetric Analysis

Thermogravimetric analysis was performed on a TA instruments Q500 (TA Instruments, New Castle, DE, USA). The experiments were performed at atmospheric pressure. A constant flow of inert gas (nitrogen, flow rate 40 mL/min) was maintained throughout the experiment. The same amount of the aqueous sample 30 μL was loaded to a tared platinum pan via micro-pipette in order to maintain the same depth of solution. The pan used has a 9.4 mm diameter platinum pan from TA instrument, and was suspended in the furnace. In order to avoid evaporation before reaching temperature, the sample was heated to the experimental temperature 298.15 K at 100 K/min. The temperature of the sample was measured by a thermocouple placed aside the pan. The sample was held isothermally at the experimental temperature for 360 min and the weight percent change of the sample was recorded. Each CUP solution was run in triplicate. The evaporation rate is very sensitive to the exposed surface area, any uneven or damage of the pan will cause unpredicted experimental error. The handling of the pan should be done very carefully, any damage to the pan will result in the need to replace the pan and do a recalibration. Deionized water was run periodically to verify that the pan had not changed due to damage or contamination. It should also be noted that at pH 8.5 CO_2_ may be absorbed and shift the pH and also alter the composition. Avoid exposure of the solution to ambient air and check pH periodically to ensure the system has not been compromised.

## 3. Results and Discussion

### 3.1. Polymer Synthesis and Characterization

Polymers 1–6 were previously synthesized and reduced in a study defining the amount and properties of surface water by DSC [27]. The polymers selected for this study were based on particle size and surface charge density issues which have been shown to dominate the properties of surface water, viscosity, and density. The six polymers’ properties; molecular weight, polydispersity, acid number, particle size and density are given in Table 1. Polymers 1–3 had the same monomer ratio, the acid number was fairly constant, and the higher acid number for Polymer 4 is due to the higher monomer ratio of MAA. Polymer 5 has the lowest acid number because the monomer ratio is the lowest. The polydispersity indicates a relatively narrow size distribution unlike most nano particulate systems.

### 3.2. Method for Evaporation Rate Determination

TGA was used to directly measure the total mass percent loss per unit time and was then converted to the actual mass loss per unit time and then the evaporation rate was calculated by Equation (1).

(1) $$R=m\cdot \left(X_{i}-X_{i+1}\right)/\Delta t$$

where *R* is the evaporation rate of the measured sample, *m* is the mass of the sample, *X_i_* is the weight fraction at time *i*, *X_i_*_+1_ is the weight fraction at time *i* + 1, Δ*t* is the time interval.

**Figure 1:**
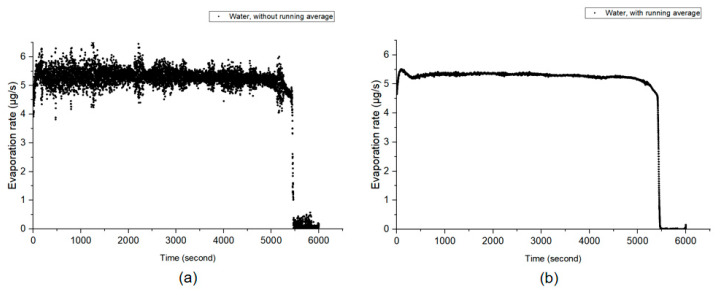
Evaporation rate of deionized water, (**a**) raw data (**b**) with running average.

The evaporation rate of deionized water was measured as the standard, shown in Figure 1. The first few hundred seconds exhibited an oscillation due to the thermal over run of the TGA and the system coming to equilibrium.

After an initial period of evaporation, the rate of mass loss of the sample remained constant with the plot of sample mass versus time resulting in a straight line. Data was collected every 0.6 s, with the very small amount of mass loss per second and a small amount of vibration, a significant amount of noise was observed in the data [26]. To reduce the noise in the TGA data, a running average method over 50 data points was used. 

**Figure 2:**
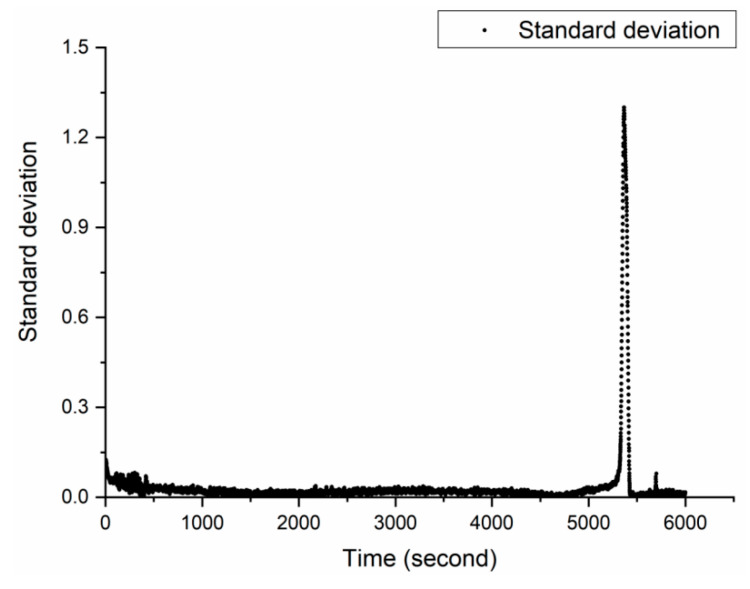
Deviation of evaporation rate of deionized water.

A plot of the standard deviation of four different samples at each data point vs time, after running average is shown in Figure 2. In the very beginning of the measurement, a large standard deviation was observed. This noise is due to surface area not being uniform initially and the temperature overshoot. Once the sample reaches a steady state the noise level drops to a very low level until about 5000 s. As the pan nears dryness the water cannot cover the entire bottom of the pan, the surface area will exhibit a large random change. The data in the last part of the measurement cannot be trusted to represent the true evaporation rate for water. During the scan, for measurements between about 500 and 5000 s, the deviations are very small, making the data more reliable with minimal scatter. 

### 3.3. Evaporation Rate of Free Water from CUPs

**Figure 3:**
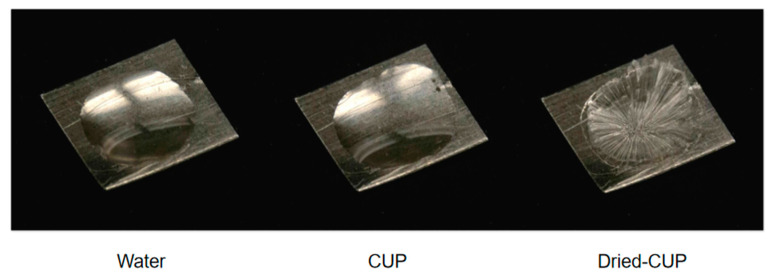
Photo of deionized water and CUP wetting and dry on platinum substrate.

The evaporation of both water and CUP solutions are highly dependent upon surface area. If the sample does not wet the platinum pan it can result in changing areas caused by the sample size and contact angle. To evaluate this, two platinum flags were cleaned, and one had 15 microliters of deionized water placed on its surface and the other had a 10% Polymer 4 CUP solution at the same volume placed on it. Figure 3 shows the image of the flags at time zero just after application and the third image after the CUP solution was dry. It can be seen that water wet the platinum partially, but the CUP solution wet much better. Once dry the CUP sample formed a relatively even coating which cracked due to poor adhesion and low crystal lattice energy. This experiment indicates that the evaporation should be representative, even to the end for CUP since it evaporates evenly. 

**Figure 4:**
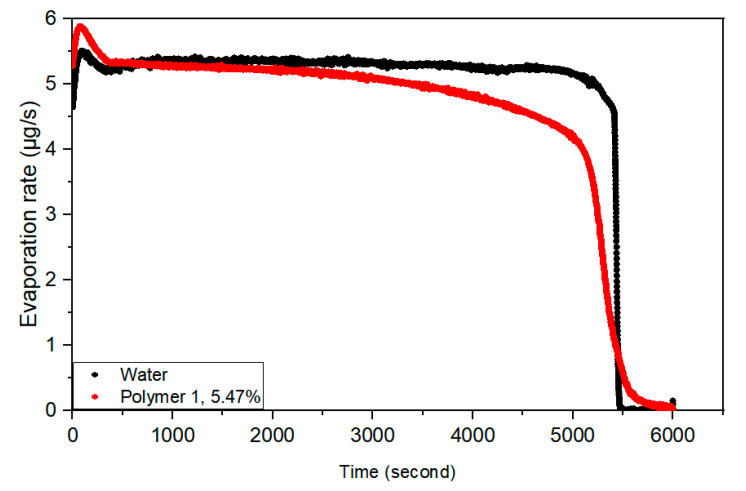
Evaporation rate of 5.47% Polymer 1 solution and deionized water.

In order to investigate the CUP particles’ effect on the evaporation rate, a 5.47% Polymer 1 solution was measured following the same protocol as with water, and compared with deionized water, as shown in Figure 4.

**Figure 5:**
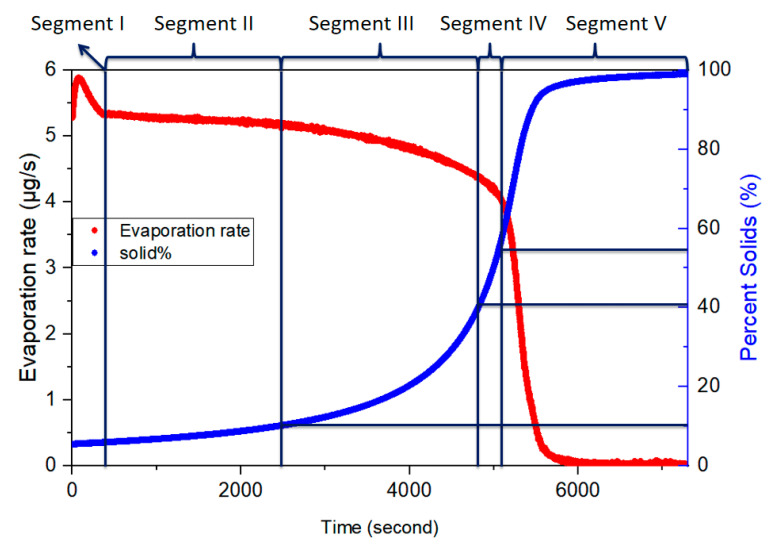
Segments I, II, III, IV and V during the isothermal process.

The Polymer 1 solution evaporated faster than deionized water in the beginning, and kept decreasing along the isothermal process, with multiple changes of evaporation rate reduction being observed. These complexities indicated that there was more than one factor involved during the isothermal process. The study separated the evaporation process into five segments designated as I, II, III, IV, and V, shown in Figure 5.

Segment I was the initial time frame of 480 s, before major compositional changes occurred. Segment II is for the range from Segment I until Manning condensation occurs. Segment III covers Manning condensation. Segment IV is the gelation of the solution and Segment V is the loss of the last water including surface water. 

**Figure 6:**
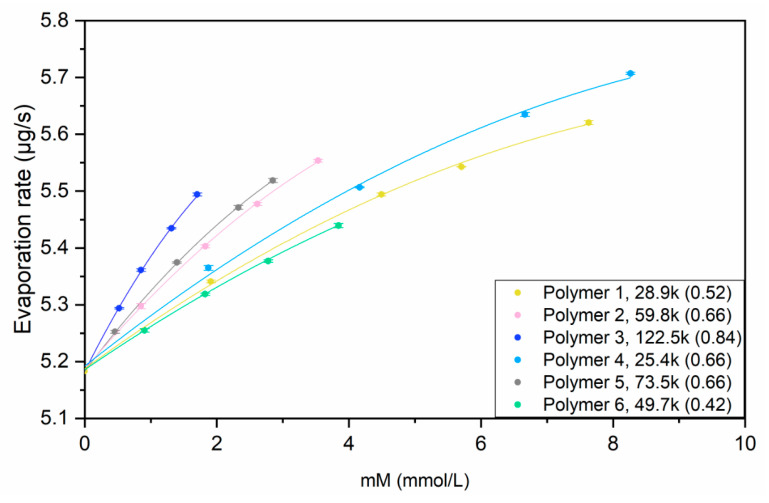
Evaporation rate of Polymer 1–6 solutions at various mM.

The evaporation rate of CUP solutions was not constant. Therefore, in order to investigate the CUP’s effect on the evaporation rate in the beginning, the evaporation rate of various polymer solutions with multiple molarities were determined immediately after the pan settled down, at 480 s (Segment I), as shown in Figure 6. 

Polymer 1, 2 and 3 have the same monomer ratio but different surface charge density and molecular weight. It was observed that, with the same molarity, polymers with higher molecular weight and surface charge density have higher evaporation rate. If comparing Polymer 2, 4 and 5, which have the same surface charge density, the polymer with larger molecular weight had a faster evaporation rate. Van De Mark et al. found that with the presence of CUP particles, the surface tension was lower than deionized water, and it was proportional to the number of charges on the particle surface [28]. The surface tension reduction was similar but smaller than that observed for typical surfactants [29]. This surface tension reduction was also observed for polyelectrolyte systems [30]. Polymer 3 evaporated faster than Polymer 2 and 1, due to more charges, which is the same for Polymers 2, 4, and 5. It was also observed that for each CUP solution, the evaporation rate was higher for the solutions with a higher initial molarity because the higher initial molarity having more charges. However, the effect of surface tension should not be a major factor for evaporation, but it will have an effect on interfacial mobility. In order to further investigate the surface effect, the relation between surface tension and the evaporation rate of aqueous salts was examined, as shown in Table 2. 

Sodium chloride was chosen since it causes an increase in the surface tension and sodium acetate, which has a carboxylate like CUP, causes a decrease in surface tension. The evaporation rate difference from water divided by the surface tension difference from water was used to evaluate the effect each had on the two, ΔR/Δγ. It was shown that, with less surface tension, sodium acetate solution evaporated slower than deionized water, due to the salts [31,32]. Also, the evaporation rate change was moderately lower than the change in surface tension for all four values. This data indicates that the primary effect on increasing the evaporation rate of CUPs is not surface tension.

Table 3 gives the surface tension and evaporation rate for CUP solutions of Polymer 1–6 at 2 mM. It should be noted that the change in the surface tension for these polymers are about 500 times higher than that for sodium acetate at the same concentration. Therefore, the molar concentration of CUP may not be a simple relationship. Table 3 also gives the number of carboxylate groups on each CUP. The number of carboxylates were partially responsible for the larger effect of both evaporation rate and surface tension. The chains of the CUP particle are not free to move and thus their relationship to each other define the “more hydrophobic” regions from the carboxylate. These hydrophobic regions are larger than those of the methyl group of the acetate ion. However, the more hydrophobic surface is dominated by the ester groups and likely some of the methyl groups of the backbone and ester. The surface tension of surfactant carboxylates becomes more effective as the aliphatic chain increases. 

The use of percent solids as well as molarity and weight fraction, *X_CUP_* are relevant to different aspects of this study Equation (2) relates these terms.

(2) $$X_{CUP}=\frac{M_{W}\cdot c\cdot 10^{3}}{\rho_{s}}$$

where *M_w_* is the molecular weight of the polymer, *ρ_s_* is the density of CUP solution, *c* is the molarity, and *X_CUP_* is the weight fraction of CUP solids.

When a very dilute CUP solution was at its equilibrium condition, the solution was homogeneous and CUP particles were randomly distributed and stabilized by the combination of Brownian motion, solvation by water and charge repulsion between particles. Assuming that each CUP occupies an average cubic volume in solution, which gives the largest distance between particles. At a given percent solids, the distance between two CUP particles was estimated by Equation (3).

(3) r=(MXCUP·ρ·NA3)−d

where *r* is the distance between two particles, *M* is the molecular weight of CUP, *X_CUP_* is the weight fraction of CUP, *ρ* is the density of solution, *d* is the size of the CUP particle, *N_A_* is Avogadro constant.

The distance between two CUP particles was determined to be from 5.5 to 8.8 nm depending on the particle size at 5% solids. The electrostatic effective distance between two CUP particles can be estimated by Equations (4)–(6) [33,34,35].

(4) $$\kappa^{-1}=\sqrt{\frac{\varepsilon_{r}\cdot \varepsilon_{0}\cdot k_{B}\cdot T}{2\cdot 10^{3}\cdot N_{A}\cdot e^{2}\cdot I}}$$

(5) $$I=\frac{1}{2}\cdot \left(M\cdot 1\cdot n_{c}\cdot 1\right)$$

(6) $$d_{eff}=2\cdot \left(\kappa^{-1}\right)$$

where *I* is the ionic strength, *M* is the molarity, *n_c_* is the number of carboxylate groups per CUP, *ɛ*_0_ is the permittivity of free space, *ɛ_r_* is the dielectric constant for water, *k_B_* is the Boltzmann constant, *e* is the elementary charge, and *κ^−^*^1^ is Debye length.

**Figure 7:**
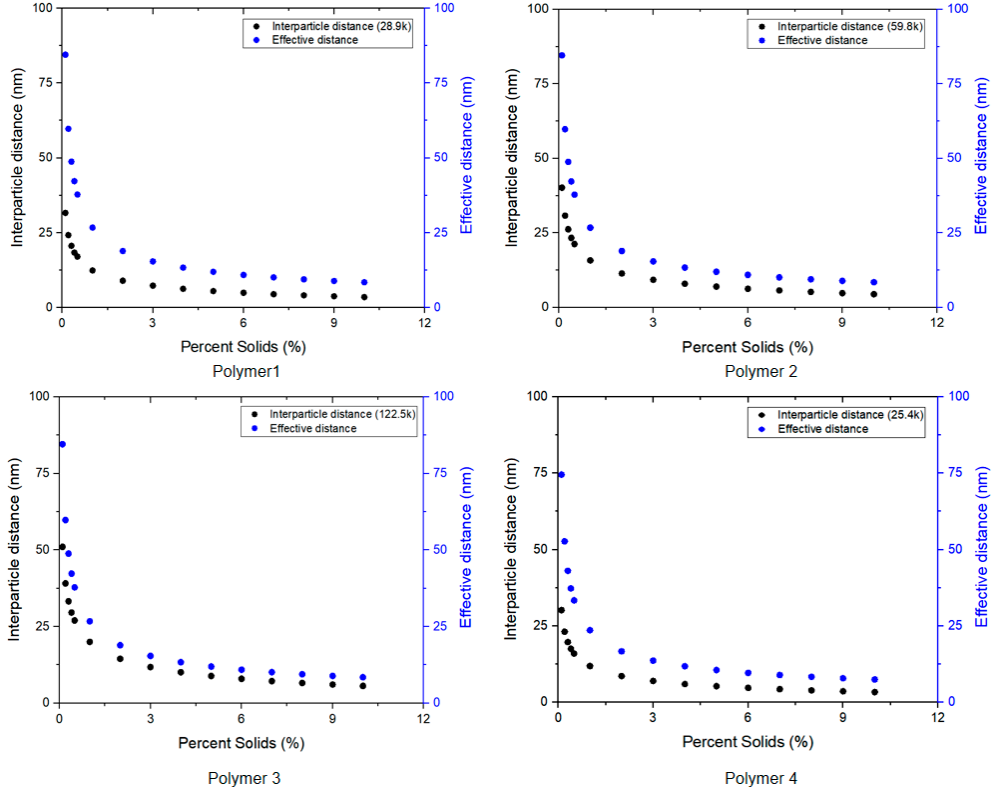
Comparison of effective distance and inter-particle distance for Polymer 1–4.

The assumption is that we have a single point charge separated by water. As Figure 7 shows, the effective distance was always larger than the estimated inter-particle distance, which indicated that the electrostatic repulsion force occurred at a CUP concentration of 1% and higher. At a constant percent solids, the CUP with smaller particle size tends to have a larger difference between the effective distance and inter-particle distance, due to a higher number of particles that results in a higher repulsion force. Polymer 1 has a higher effective distance to inter-particle distance ratio than Polymer 3, because of a larger number of particles at the same percent solids.

**Scheme 1:**
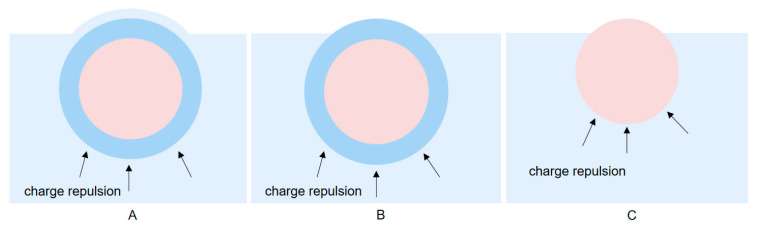
Deformation of water surface at air-water interface by CUP particles due to charge repulsion. (**A**–**C**) represent the three possibilities when CUP particles penetrate water-air interface due to the charge repulsion.

Due to coulomb’s law, the repulsion force is proportional to *1/r^2^* [36], where r is the distance between two charges. Consider each CUP particle as a point charge, and assume an r value of 9 nm, the electrostatic repulsion force for Polymer 1 is 2.85 × 10^−12^ N, while the surface tension of water is 7.22 × 10^−11^ N/nm and surface tension for 5.47% Polymer 1 solution is 7.08 × 10^−11^ N/nm. Since each CUP particle has multiple charges (29.3 to 124.1 charges per particle for Polymers 1 and 3 respectively), the actual repulsion force was expected to be much larger than 2.85 × 10^−12^ N. Therefore, at 5% solids, the charge repulsion between CUP particles should be strong enough to cause deformation of the air-water interface as shown in Scheme 1.

**Scheme 2:**
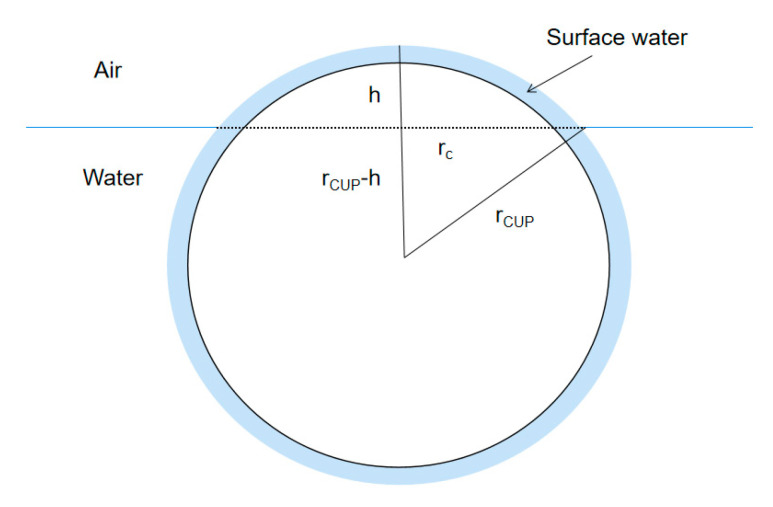
Deformation of water at air-water interface by CUP particles due to charge repulsion.

The three models indicate A: CUP with surface water and a layer of air/surface water, B: CUP with a layer of surface water, and C: Cup particle with no water. Model C can be eliminated, because CUP particles are highly hydrophilic on the surface, and have a layer of strongly associated surface water [27]. If model C were the case, all the evaporation rate would be due to edge effects on the surface tension and the loss of surface area occupied by the CUP particles would reduce the evaporation rate. Therefore, with the presence of CUP particles, the interface water deformed causing a decrease in surface tension, according to the Gibbs isotherm, and an increase in surface area. Assuming all the observed increase in the evaporation rate were contributed by increased surface area at the interface, the increased ratio of evaporation rate should be proportional to the increased surface area. At a given percent solids, the degree of interface water deformation (Scheme 2) could be calculated by Equation (7).

(7) h=ΔRR·π·(Mwρ·XCUP3)

where *h* is the height of the interface water deformation, Δ*R* is the increased evaporation rate compared with water, *R* is the evaporation rate of the CUP solution, *M_w_* is the molecular weight of CUP, *ρ* is the density of the solution, *X_CUP_* is the weight fraction of CUP.

The *h* was calculated to be from 0.70 to 1.27 nm with the range of about 5 to 20% solids, *h* depends on the percent solids and molecular weight of CUP particles. The particle size of CUPs ranges from 4.02 to 6.83 nm, due to the moderate repulsive force, interfacial water deformation may be the major contributor. The surface tension on the deformational region may be lower than normal water and the circumference region will have a significantly lower surface tension. It is most likely that Model A or B is the correct one and that contribution from the increased surface area is the cause of the enhanced evaporation at low concentrations. It is well known that polymers in solution reduce the vapor pressure of the solvent. This would in general reduce the evaporation rate of a CUP solution. The increased area must overcome this small reduction in vapor pressure also.

In Segment I, the main factor that dominated the evaporation rate was the increased surface area which increased the rate. At a given molarity, the CUP with larger particle size has shorter inter-particle distance, resulting in a higher charge repulsion force, that increases the amount of interfacial water deformation, h. In addition, more charges on the CUP surface caused more surface tension reduction, the combination of these two effects showed a higher evaporation rate. Looking back to Figure 4, 25.4k, Polymer 4 has a similar particle size as 28.9k, Polymer 1 and the CUP surface tension was lower for Polymer 4 and the interfacial deformation was greater due to the larger charge repulsion, therefore it showed a higher evaporation rate. Polymer 6, 49.7k has the lowest charge density but a higher molecular weight, particle size, than Polymer 1. The surface tensions for Polymer 1 and 6 are similar and the evaporation rate for Polymer 6 is slightly higher. This is because with the same molarity, Polymer 6 has a shorter inter-particle distance due to the larger particle size, that resulted in a higher charge repulsion. The interfacial water deformation for Polymer 6 would be expected to be greater thus showing a higher evaporation rate. 

As water continuously evaporated during the isothermal process, the temperature at the interface decreased due to the lost heat of vaporization and the surface molarity/percent solids of CUP particles at the interface became higher than the bulk solution, Segment II. As the surface molarity/percent solids increases, it sets up an osmotic gradient with the bulk solution. The osmotic gradient draws water to the surface to dilute the CUPs [37,38]. The movement of water to the surface not only dilutes the CUP at the air interface but also brings heat to reestablish equilibrium. At the same time, CUP particles experience a higher charge repulsion and move toward the bottom through translational diffusion at low concentration. The reduced temperature at the interface decreases the evaporation rate, and the increased molarity provided higher charge repulsion to create an increase in the interfacial water deformation that will increase the water evaporation rate. However, the evaporation rate largely depends on the diffusion rate of water molecules to the interface [11]. The viscosity in the interfacial region will be increasing with the increasing of CUP molarity/percent solids, due to the secondary electroviscous effect [39], which was demonstrated by Van De Mark et al. [40]. The increased viscosity slowed movements of both the water and CUP particles, which explains a slower observed evaporation rate.

The diffusion coefficient could be determined by Stokes–Einstein equation [41].

(8) $$D=\frac{K_{B}\cdot T}{6\cdot \pi \cdot \eta \cdot r}$$

where *K_B_* is Boltzmann constant, *T* is the absolute temperature, *η* is the viscosity of the solution, and *r* is the radius of CUP particle.

Table 4 gives the diffusion coefficient for the six polymers at 5% and 10% at 298.15 K. The lower diffusion coefficient at higher concentrations is due to charge repulsion increasing the viscosity and drops faster as the concentration increases approaching infinity at the gel point.

**Figure 8:**
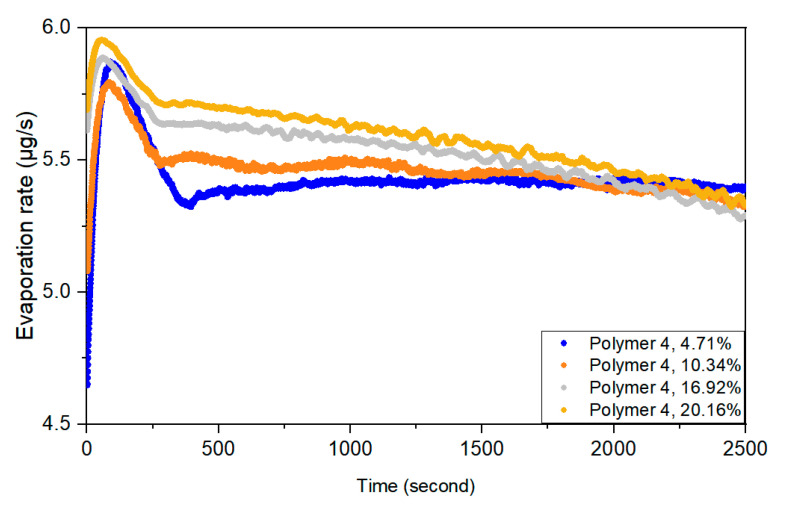
Evaporation rate of Polymer 4 solutions at 4.71%, 10.34%, 16.92% and 20.16% in the first 2500 s.

The evaporation rate of Polymer 4 solution with initial percent solids of 4.71%, 10.34%, 16.92% and 20.16% in the first 2500 s were evaluated as an example, shown in Figure 8.

**Figure 9:**
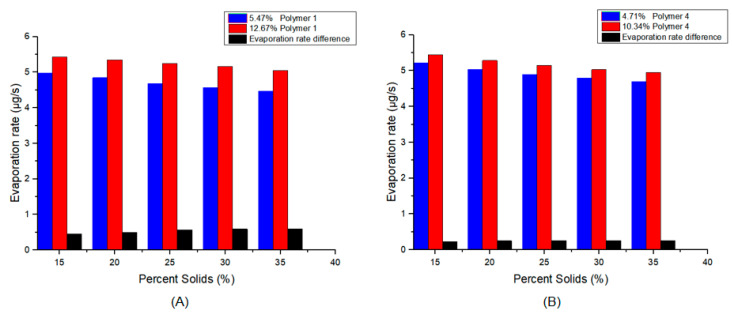
Evaporation rate of Polymer 1 (**A**) and Polymer 4 (**B**) solutions with different initial %solids concentrations.

It was shown that, the higher initial percent solids of CUP solution, the faster the initial evaporation rate was, due to the increased surface area, Segment I. However, when the water started to evaporate, the evaporation rate of all CUP solutions decreased, except the 4.71% which retained its evaporation rate for a longer time before decreasing. The change was more obvious for higher initial percent solids CUP solutions. Because the higher percent solids solution has higher viscosity, that resulted in a slower movement of particles and water molecules in Segment II. The 4.71% solution was dilute enough that the translational diffusion and osmotic flow kept the surface CUP concentration lower for a longer time as the evaporation progressed. The low mobility of particles and water molecules further enhanced the CUP particles stacking at interface, increasing the viscosity, and reducing the evaporation rate. This observation was further investigated by comparing the evaporation rate when two different initial percent solids solution were evaporated to the same percent solids. The evaporation rate of Polymer 1 and Polymer 4 solutions with different initial percent solids were evaluated during the drying process at the same solids content, as shown in Figure 9.

It was seen that when concentrated to the same percent solids, the evaporation rate of the low initial percent solids solution was lower. The low solids sample must first loose significant water which creates a higher concentration of CUP particles at the surface which increases the viscosity lessening the diffusion of water to the surface and slowing the movement of CUP particles away from the surface as well as lowering the surface temperature. For the lower concentration the total solution thickness is decreasing with time as does the higher concentration, however the rate of change in the thickness is almost twice as much for the lower concentration. The shorter distance will also influence the result by reducing the osmotic flow since the liquid thickness increases the opportunity to set up osmotic gradients. Polymer 1 shows a greater difference in evaporation rate than Polymer 4. The rate differences may be due to the higher charge density of Polymer 4 forcing the particles to rearrange positions more rapidly and increasing viscosity. Polymer 4 has a slightly lower mass which makes the charge effect even more meaningful.

As evaporation progressed in Segment II, the movement of water molecules and CUP particles caused by osmotic pressure and charge repulsion were the dominant driving forces in this segment. As the concentration increased the viscosity reduced the osmotic flow which in turn allowed the temperature of the surface to fall lower since the rate of warm water being transferred to the surface slowed.

The ionic force between particles forces CUP particles down from the surface by each one pushing down the particle below it, which helped to minimize osmotic differences. The ionic force also increases the vertical displacement of the CUP particles at the air interface as the CUP concentration increases. The air surface area with free water decreases and the area of CUP surface water increases and dominates evaporation as the solids content rises. When the concentration at or near the surface hits about 20% solids the CUP surface ions begin to undergo Manning type condensation which lowers the effective charge. When this condensation begins to occur the CUP particles can increase their packing concentration and reduce the repulsion on their neighbor as well as limit the CUP penetration through the air interface.

**Figure 10:**
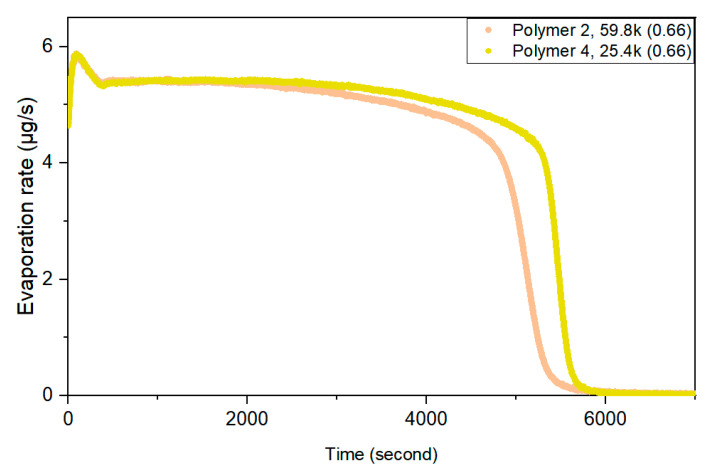
Evaporation rate of Polymer 2 (1.83 mmol/L) and Polymer 4 (1.87 mmol/L).

In addition, particle size is another important factor, because it directly influences the diffusion rate of CUP particles. The Polymer 2 and Polymer 4 solutions with similar molarity were compared, as shown in Figure 10.

It was seen that, in Segment I, the Polymer 2 solution evaporated slightly faster due to more charges per particle than Polymer 4. However, Polymer 2 solution started to show a larger and larger evaporation rate reduction as compared to Polymer 4 solution through Segment II. This reduction was due to a larger particle size diffusing slower, which caused more particles to stack up at the air-water interface. This also increased the viscosity at the interface, and further decreased the diffusion rate of particles, and therefore, Polymer 2 presented a slower evaporation rate through Segment II.

Previous studies have demonstrated that when the percent solids increased above 20%, inter-molecular counterion condensation occurred, segment III [27]. Increasing the CUP percent solids also increased the counterion concentration, which condensed on the CUP surface reducing its effective charge [29]. The phenomenon known as Manning condensation (counterion condensation) is widely accepted in charge stabilized colloidal suspensions [42]. The inter-molecular counterion condensation causes the effective charge to be lower than the bare surface charge and allows more CUP particles with better packing at the air-water interface. At the same time, the total number of charged groups at the air-water interface increases because only a small fraction of the charged groups on the CUP surface undergo Manning condensation. The inter-molecular counterion condensation decreases the charge repulsion effect to a degree, therefore, decreases the mobility of CUP particles to the bottom as a result of charge repulsion.

**Figure 11:**
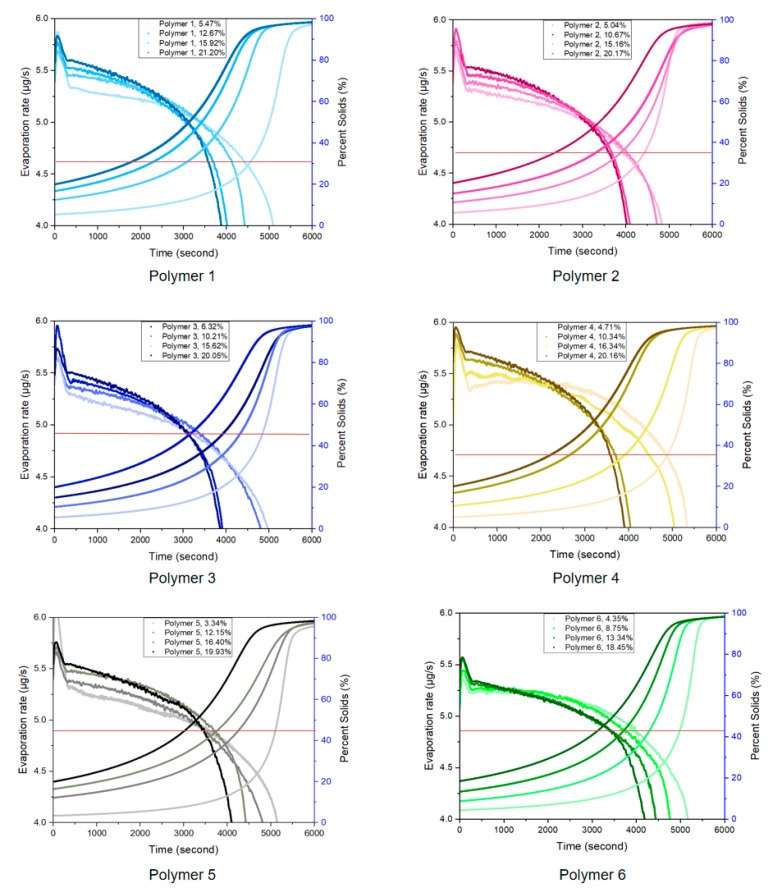
Evaporation rate at various percent solids for Polymer 1–6.

Using Polymer 6 as an example, it was shown that with the increasing of the initial percent solids, the inter-molecular counterion condensation occurs earlier in the time frame. This drop in the rate can be observed in Figure 11. The darker line drops to an evaporation rate of 4 micrograms per second first while the 4.35% occurs much later. Segment III begins with inter-molecular counterion condensation and ends with gelation as random close packing, RCP, which is marked with a red line in Figure 10. Starting at low concentration it requires more time to reach RCP as well as the start of Manning condensation. Once gelled, the CUP particles cannot move translationally, but they can move as a unity, shrinking all the spaces between particles uniformly to avoid significant stress development. Rapid evaporation has been noted to cause crack development in a drying sample with the surface shrinking due to drying before the system can reestablish equilibrium.

The evaporation of water, as it approaches the end of Segment III, slowed as the percent solids of the bulk solution increased, this reduced the distance between particles in the bulk portion. The particles reached a pseudo random close packing state which was defined as the gel point of CUP, and then with a small additional loss of water became pseudo hexagonal close packing (HCP), Segment IV. The term pseudo HCP was used since the particles have a distribution of diameters and charges so it will not be a perfect HCP lattice. As the particles formed an organized structure where each particle occupied a lattice position even in the bulk solution, the mobility of water molecules and CUP particles were highly limited [40]. Thus, the evaporation rate decreased even faster. All water diffusion was either through the CUP surface water or through the voids between the spheres occupied by free water with the state of surface deformation having little meaning since the surface is now occupied by CUP particles with their surface water only.

### 3.4. Evaporation of CUP Surface Water

**Scheme 3:**
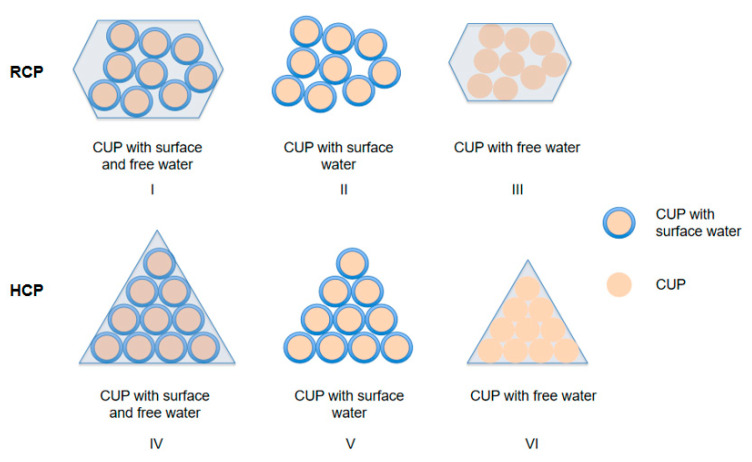
Possibilities during surface water evaporation process.

As water molecules continuously escaped from the interface, the particles approach each other, and the increased electrostatic repulsion tends to arrange them in positions with equal distance from the nearby particles. There are two types of packing models for spheroidal materials when the percent solids are very high, random close packing and hexagonal close packing. CUP particles will first approach random close packing as the concentration increases and slowly, through movements driven by the repulsive forces between particles approach hexagonal close packing. At this point the particles are only surrounded by surface water with a small amount of free water occupying the space in the voids, Segment IV. Many previous studies have shown that surface water has a much lower mobility, higher density [43], and tighter association with the hydrophilic groups [44]. Therefore, the evaporation rate of surface water is expected to show very different behavior from free water. The viscosity of the solution is close to infinity at this point and free water has to move primarily through the surface water layer. The rate of water diffusion is therefore very small and significantly reduces the rate of evaporation. There are potentially three possibilities for each packing model, shown in Scheme 3. CUP with surface water and free water, CUP with free water and CUP only with surface water. Previous studies have eliminated models III and VI since the existence of a surface water layer has been demonstrated [45].

In order to evaluate the evaporation process of the surface water and the last trace of free water in the voids, the percent solids of CUP particles for each packing model can be calculated by knowing the max volume fraction of random close packing, which is 0.634, and 0.7405 for hexagonal close packing [46,47,48,49,50], according to Equations (9)–(11).

(9) $$\phi =\frac{\rho_{s}\cdot X_{CUP}}{\rho_{p}}$$

(10) $$\phi_{R-\max}{\left(1+\lambda /r\right)}^{3}=0.634$$

(11) $$\phi_{H-\max}{\left(1+\lambda /r\right)}^{3}=0.7405$$

where *ϕ* is the CUP volume fraction, *ρ_s_* is the density of CUP solution, *ρ_p_* is the density of CUP particle, *ϕ*_*R*-max_ is the maximum volume fraction for random close packing, *ϕ*_*H*-max_ is the maximum volume fraction for hexagonal close packing, *λ* is the thickness of surface water, and *r* is radius of the CUP particle.

The percent solids of CUP for the possibilities of each were calculated and are shown in Table 5.

**Figure 12:**
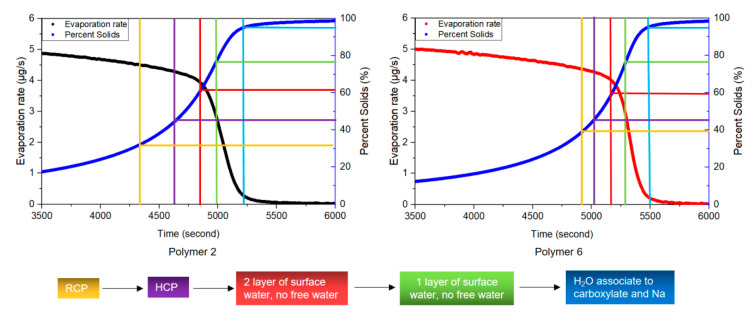
Evaporation rate of water at different percent solids, 5.04% Polymer 2, 4.35% Polymer 6.

The amount and thickness of CUP surface water has been determined by DSC [27]. As it was discussed in Figure 1, for the last water loss, a large reduction in the evaporation rates were observed near the end. One possible reason for this issue was an insufficient amount of solution to cover the pan bottom. In this case, 5.04% Polymer 2 and 4.35% Polymer 6 solutions were used as examples. Knowing the diameter of the pan being 9.4 mm, it was calculated that even when there is only CUP solids existing in the pan, the bottom of the pan is still fully covered with a 0.02 mm depth. By applying the results from Table 5 to the evaporation curve, Scheme 1 indicates that CUP solutions dry relatively uniform wetting the platinum pan. The CUP solution surface tension decreases with increasing concentration making it more wetting of the pan. Therefore, in Segment V, the evaporation rate reduction was not because of the lack of sufficient solution to cover the pan. Figure 12 shows both the evaporation rate of Polymer 2 and 6 solution and their corresponding percent solids.

By comparing the percent solids of CUP at each slope change of the evaporation rate curve with the results in the Table 2, we illustrate the process of how surface water and free water in the voids evaporate. The results showed that evaporation rate sharply decreased when the percent solids passes the HCP, due to the highly limited mobility of particles and water molecules. Another big evaporation rate reduction occurred at about 53%, where there is no free water between the CUP particles and two layers of surface water around CUP particles, that implied surface water doesn’t evaporate until all free water is released. The next step was at about 72% solid, where there was only one layer of surface water, due to the inner layer being more tightly hydrogen bounded to the CUP surface. Furthermore, at about 96% solid, there was another evaporation rate change.

The results imply that free water completely evaporated before surface water started to evaporate, and water molecule associated to carboxylate group are released in the end. This was demonstrated for CUP solutions with different molecular weight and surface charge density, and indicated it is molecular weight and surface charge density independent.

Sodium acetate hydrate has three waters of hydration which are held relatively strongly. Based on Table 4, Polymer 2 and Polymer 4 should have 94.92% and 96.55% solids respectively if it had 3 waters of hydration also. As can be seen in Figure 11 the two polymers are very close to these values. Therefore, it is highly likely that the last three waters to leave are those associated with the surface carboxylates.

## 4. Conclusions

The TGA method is a useful screening method for evaporation rate measurements with only small amounts of substance required. The experiments are quick and easy and yet provide accurate results for comparing different substances. Results indicated that CUP was able to cause interfacial water deformation due to inter-particle charge repulsion, which increases the surface area and reduces surface tension, that increases the evaporation rate. In addition, the viscosity, in other words the mobility of CUP particles and water molecules, is also an important factor for the evaporation rate. The CUP solution with higher initial percent solids has a higher evaporation rate in the beginning of the isothermal process than deionized water, due to more interfacial deformation and increased surface area as a result. During the isothermal process, the evaporation rate decreased, because of the combination of the effect of a decrease in air–water interface temperature and limited mobility of water molecules and CUP particles by the increased viscosity. When reaching RCP and HCP, the movement of free water molecules were highly retarded, that caused significant evaporation rate reduction. Surface water didn’t evaporate until all free water evaporated, and presented a slower evaporation rate. Water molecules associated with the carboxylate groups on the CUP surface evaporated last.

## Figures and Tables

**Table 1:** Molecular weight, particle size, acid number and density of the polymers [27].

Sample ID	M_n_/PD (g/mol)	Monomer Ratio	Particle Size (nm)	AN (mg KOH/g)	Density of Dry CUP, ρ_p_ (g/mL)	Charge Density in Ions per nm^2^, ρ_v_
Polymer 1	28.9k/1.8	9:1	4.22	56.8	1.2246 ± 0.0018	0.52
Polymer 2	59.8k/1.7	9:1	5.38	57.0	1.2311 ± 0.0014	0.66
Polymer 3	122.5k/1.7	9:1	6.83	56.9	1.2342 ± 0.0018	0.84
Polymer 4	25.4k/2.3	6.8:1	4.04	73.2	1.2243 ± 0.0018	0.66
Polymer 5	73.5k/1.7	9.8:1	5.76	52.6	1.2315 ± 0.0018	0.66
Polymer 6	49.7k/1.8	14:1	5.06	37.7	1.2307 ± 0.0016	0.42

**Table 2:** Comparison of surface tension and evaporation rate of sodium salts.

	Water	NaCl	NaCl	NaAc	NaAc
Concentration	0	0.35M	0.89M	1M	2M
γ (mN/m)	72.2	73.9	75.6	70.2	69.2
Δγ	0	+1.7	+3.4	−2.0	−3.0
R (μg/s)	5.18	5.09	4.95	5.04	4.99
ΔR	0	−0.09	−0.23	−0.14	−0.19
׀ΔR/Δγ׀	N/A	0.05	0.07	0.07	0.06

**Table 3:** Comparison of surface tension and evaporation rate of CUP solutions.

	Polymer 1 (28.9k) 0.52	Polymer 2 (59.8k) 0.66	Polymer 3 (122.5k) 0.84	Polymer 4 (25.4k) 0.66	Polymer 5 (73.5k) 0.66	Polymer 6 (49.7k) 0.42
Molarity(mmol/L)	2	2	2	2	2	2
γ (mN/m)	70.9	68.4	66.6	68.5	68.5	71.0
Δγ	−1.3	−3.8	−5.6	−3.7	−3.7	−1.2
R (μg/s)	5.32	5.42	5.548	5.37	5.44	5.34
ΔR	+0.16	+0.24	+0.37	+0.19	+0.26	+0.14
׀ΔR/Δγ׀	0.12	0.06	0.07	0.05	0.07	0.12
n*acid	29.3	60.6	124.1	33.1	68.9	33.4
n*acid/n*ester (per CUP)	0.232	0.310	0.418	0.296	0.305	0.183

Notes: γ is surface tension, R is evaporation rate. Δγ is the surface tension difference between water and the CUP solution, ΔR is the difference in evaporation rate for water vs. CUP solution.

**Table 4:** Diffusion coefficient of CUP particles at 298.15 K (×10^−6^ cm^2^/s).

%Solids	Polymer 1 28.9k	Polymer 2 59.8k	Polymer 3 122.5k	Polymer 4 25.4k	Polymer 5 73.5k	Polymer 6 49.7k
5%	1.59	1.16	0.74	1.69	1.10	1.31
10%	1.15	0.83	0.45	1.24	0.78	0.92

**Table 5:** Percent solids for CUP Polymer 2&6 for HCP and RCP.

Possible Packing	2 Layer of Surface Water with Free Water	1 Layer of Surface Water with Free Water	2 Layers of Surface Water	1 Layer of Surface Water	Solids Only	Three Water Molecules per Carboxylate Group
RCP (59.8k)	32.56	45.53	60.94	77.47	100	94.92
HCP (59.8k)	46.71	54.90	60.94	77.47	100	
RCP (49.7k)	39.50	51.31	59.39	76.42	100	96.55
HCP (49.7k)	45.84	58.92	59.39	76.42	100	
